# Small molecules that inhibit Vif-induced degradation of APOBEC3G

**DOI:** 10.1186/1743-422X-11-122

**Published:** 2014-07-01

**Authors:** Masashi Matsui, Keisuke Shindo, Taisuke Izumi, Katsuhiro Io, Masanobu Shinohara, Jun Komano, Masayuki Kobayashi, Norimitsu Kadowaki, Reuben S Harris, Akifumi Takaori-Kondo

**Affiliations:** 1 Department of Hematology and Oncology, Graduate School of Medicine, Kyoto University, 54 Shogoin-Kawaracho, Sakyo-ku, Kyoto 606-8507, Japan; 2 Japanese Foundation for AIDS Prevention, Tokyo 101-0061, Japan; 3 AIDS Research Center, National Institute of Infectious Diseases, Tokyo 162-8640, Japan; 4 Department of Biochemistry, Molecular Biology and Biophysics, Minneapolis, USA; 5 Institute for Molecular Virology, University of Minnesota, Minneapolis, MN 55455, USA

**Keywords:** HIV-1, Vif, APOBEC3G, Small molecules

## Abstract

**Background:**

HIV-1 Vif is essential for virus replication in natural target cells such as T cells and macrophages. Vif recruits a ubiquitin ligase to degrade restrictive APOBEC3 proteins. APOBEC3G is one of the most potent retroviral restriction factors targeted by Vif and, as such, the Vif-APOBEC3G interaction has emerged as a promising HIV-1 therapeutic target.

**Methods:**

20,000 small molecules were used in live-cell screens for those that preserve EGFP-APOBEC3G fluorescence and luciferase-APOBEC3G luminescence in the presence of HIV-1 Vif.

**Results:**

2 compounds with similar core structures preserved APOBEC3G levels in the presence of Vif. 10 μM of compound restored APOBEC3G to levels sufficient for incorporation into *vif*-proficient virus particles and restriction of virus infectivity. Vif-dependent APOBEC3G polyubiquitination and general proteasomal activity were unaffected at the same concentration.

**Conclusions:**

The small molecules described here preserve APOBEC3G levels and activity in the presence of Vif. These molecules are starting points for further development as antiretrovirals.

## Introduction

Anti-retroviral therapies for patients with HIV-1 infection have been greatly improved. Current therapies comprised of combinations of three or more drugs can suppress viral loads below detection threshold of clinical tests for many years [1,2]. These therapies must be taken continuously for the entire life of the patient because they do not eliminate integrated proviruses. This regimen unfortunately can result in a variety of adverse long-term side effects including renal dysfunction, osteoporosis, and cardio-vascular diseases [2,3]. All approved drugs for HIV-1 infection, except CCR5 antagonists, are designed to target viral enzymes, reverse transcriptase, protease and integrase, and drugs that target accessory proteins have yet to be developed for clinical use [2].

HIV-1 Vif is essential for viral replication in T cells and macrophages [4,5]. Vif protects HIV-1 from restriction by cellular APOBEC3 proteins [6-8]. Vif forms a ubiquitin ligase complex with core-binding factor beta, cullin 5, RING-box protein 2, elongin B, and elongin C, which binds APOBEC3 proteins and promotes their poly-ubiquitination and proteasomal degradation [9-13]. In the absence of Vif protein, APOBEC3 can be incorporated into budding virions and mutate viral genome during the process of reverse transcription in target cells (reviewed in [14]). APOBEC3 proteins are a family of DNA cytosine deaminases which converts cytosines to uracils in single-strand DNA [15-17]. Of these, APOBEC3D, APOBEC3F, APOBEC3G, and APOBEC3H are capable of restricting HIV-1 in T cells [18]. Over-expression of APOBEC3G alone is sufficient for suppressing virus replication in T cell-based spreading infection experiments [18]. Thus, Vif-APOBEC3G interaction has been recognized as a novel therapeutic target for patients with HIV-1 infection [19]. The disruption of any of the steps required for Vif function is expected to recover cellular APOBEC3G levels and enable virus restriction. This concept was shown originally by identifying a small compound using a fluorescence-based screen of a library of small compounds [20]. To further validate this concept and identify additional candidate Vif-inhibitors, we have screened a library of 20,000 small compounds for recovering APOBEC3G expression levels in the presence of Vif, using EGFP- and luciferase-based APOBEC3G expression systems. Here we report two new molecules that elevate APOBEC3G levels in the presence of Vif. Low concentrations of these compounds result in a suppression of HIV-1 infectivity in the presence of both APOBEC3G and Vif.

## Results

### Two small molecules which recover APOBEC3G expression in the presence of HIV-1 Vif were identified by a multi-round screening

**Figure 1:**
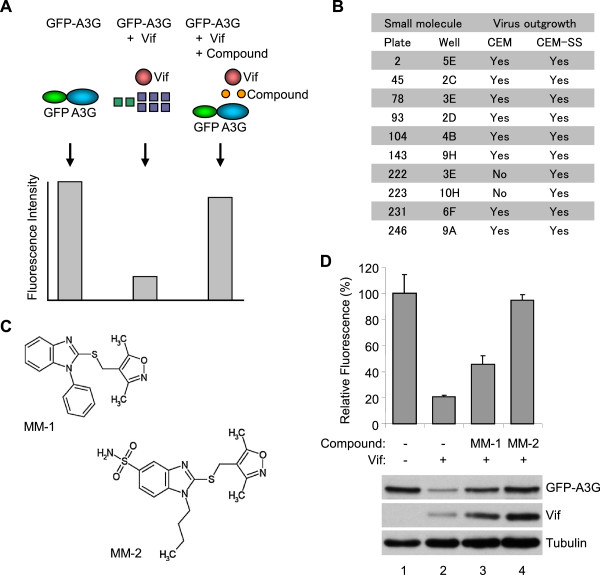
**Two candidate molecules for inhibiting Vif-mediated degradation of APOBEC3G were identified by a multi-round screening. A.** Schematic depicting of the primary screening for Vif inhibitors using EGFP-fused APOBEC3G. **B.** Spreading infection of HIV-1 in the presence of the candidate small molecules. **C.** Structures of two candidate molecules share similar backbone. **D.** Fluorescence measurement (histogram) and immunoblot analyses (panels) of the effect of two candidate molecules on EGFP-fused APOBEC3G protein levels in the presence of Vif protein. 293 T cells transiently expressing EGFP-APOBEC3G in the presence of transient expression of Vif were treated with 10 μM MM-1 or MM-2 for 24 hours. Fluorescence intensity was measured and that of DMSO-treated cells without Vif expression was normalized to 100%. Average and standard error of three independent experiments are shown in the histogram.

To screen for small molecules that recover APOBEC3G expression in the presence of HIV-1 Vif, we established a high-throughput assay using EGFP-fused APOBEC3G. 293 T cells were co-transfected with EGFP-fused APOBEC3G (EGFP-APOBEC3G) and a Vif expression vector or an empty vector as a control. As described in many prior reports, co-expression of Vif impairs APOBEC3G expression, which can be quantified using a fluorescence plate reader. If a small compound reduces the capacity of Vif to downregulate EGFP-APOBEC3G, higher fluorescence intensity will be detected (Figure 1A). We used this assay to screen a small compound library, and found 37 out of 20,000 compounds repeatedly recover fluorescence intensity more than 50%.

In the process of this screening, we noticed that if a compound itself has fluorescence, it gives a false positive result in the assay. To exclude such molecules, we performed a secondary round of screening using a luciferase-fused APOBEC3G (Luc-APOBEC3G). Similar to the primary screen, 293 T cells were co-transfected with Luc-APOBEC3G and the Vif expression vector or the empty vector as a control, and APOBEC3G expression was quantified by enzyme-based chemiluminescence assays in the presence of candidate small molecules. We found that ten out of 37 compounds recovered luminescence intensity more than 50%.

To determine whether any of these candidate molecules actually inhibit the viral replication, we then performed spreading infection experiments with these small molecules using non-permissive CEM cells, which express APOBEC3G sufficient to restrict *vif*-deficient HIV-1, and permissive CEM-SS cells, which express less APOBEC3G. We found that two out of ten small molecules appeared to inhibit the viral replication in CEM cells, but not in CEM-SS cells (Figure 1B), and called these molecules MM-1 and MM-2. Interestingly, these two compounds share similar backbone (Figure 1C). To confirm the effects of MM-1 and MM-2 on APOBEC3G expression in the presence of HIV-1 Vif, we performed immunoblotting analyses for APOBEC3G as well as measuring the fluorescence intensity. 293 T cells were transfected with the expression vector for EGFP-APOBEC3G in the absence or presence of co-transfection of the expression vector for Vif, and treated with 10 μM of MM-1 or MM-2. Co-transfection of Vif caused a reduction in fluorescence intensity, and treatment with MM-1 or MM-2 caused recovery of fluorescence intensity (Figure 1D, histogram), consistent with our previous results of the primary screening. Of note, 100 μM MM-1 or MM-2, or 1% DMSO alone does not cause fluorescence at detectable levels (data not shown), suggesting the differences of fluorescence intensity we observed are not due to inherent fluorescence of the small molecules. Consistent with fluorescence studies, immunoblotting analyses showed that treatment with MM-1 or MM-2 significantly recovered APOBEC3G protein levels (Figure 1D, panels).

### The small compounds increased A3G expression in dose dependent manners

**Figure 2:**
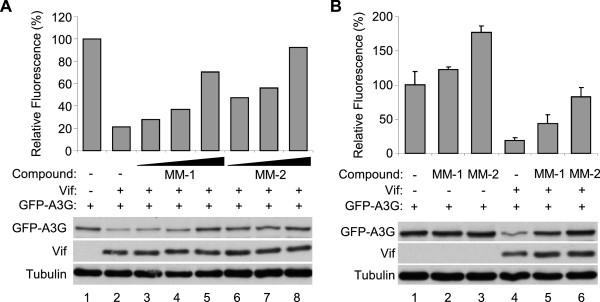
**Detailed analyses of the candidate molecules. A.** Titration of the two compounds. Treatment of MM-1 or MM2 recovered fluorescence intensity and protein levels of EGFP-fused APOBEC3G in a dose-dependent manner. 293 T cells were co-transfected with EGFP-APOBEC3G and Vif expression vectors and treated with different concentrations of MM-1 or MM-2. Fluorescence intensity was measured and that of DMSO-treated cells without Vif expression was normalized to 100%. Cell lysates were also analyzed by immunoblotting (panels). **B.** Vif dependency of the compounds. 293 T cells were transfected with EGFP-APOBEC3G expression vector in the absence or presence of co-transfection of Vif expression vector, and treated with 10 μM MM-1 or MM-2 for 24 hours. Cells were analyzed similarly to **A**, average and standard error of two independent experiments are shown in histogram.

We next performed a series of dose response experiments. 293 T cells were transfected with the expression vector for EGFP-APOBEC3G in the absence or presence of co-transfection of the expression vector for Vif, and treated with 5, 10, or 20 μM of MM1, or 2.5, 5, or 10 μM of MM-2. The treatment of MM-1 or MM-2 caused recovery of fluorescence intensity in a dose-dependent manner (Figure 2A, histogram). Consistent with these results, APOBEC3G protein levels were also recovered by MM-1 or MM-2 treatment, while the levels of tubulin were comparable (Figure 2A, panels).

### The effects of the small molecules on APOBEC3G levels were greater in the presence of Vif

We next asked whether the effects of the small molecules are dependent on APOBEC3G-Vif interaction. 293 T cells were transfected with the expression vector for EGFP-APOBEC3G, co-transfected with the expression vector for Vif or an empty vector, and treated with MM-1 or MM-2, or DMSO as control. In the fluorescence study, treatment with MM-1 or MM-2 appeared to increase APOBEC3G levels both in the absence and presence of Vif protein, but the effects were greater in the presence of Vif protein than those in the absence of Vif (Figure 2B, histogram, 2.3 fold versus 1.2 fold for MM-1, 4.3 fold versus 1.8 fold for MM-2). In the immunoblotting analyses, treatment with MM-1 or MM-2 dramatically recovered protein APOBEC3G levels in the presence of Vif, but A3G levels were comparable in the absence of Vif (Figure 2B, panels). These results suggest that the effects of these compounds are mostly Vif-dependent, but we cannot exclude the possibility that these compounds are also effective in the absence of Vif protein.

### The small molecules increased APOBEC3G incorporation into *vif*-proficient virus particles

**Figure 3:**
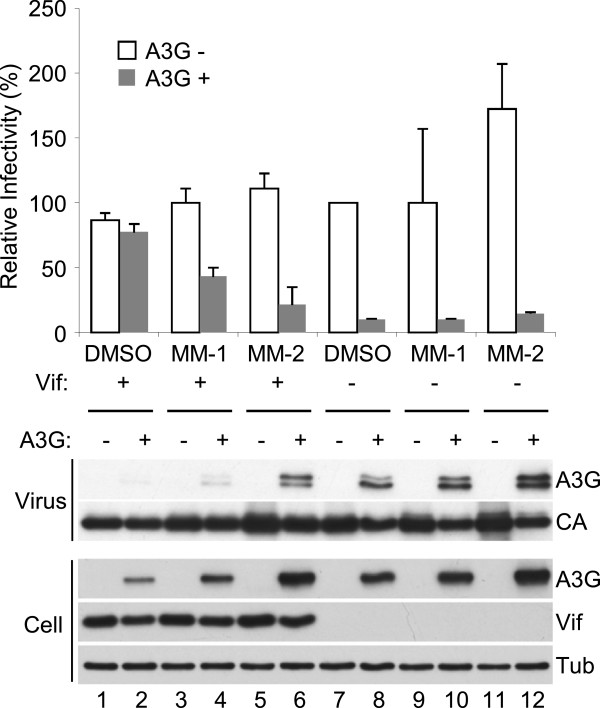
**Candidate molecules impaired virus infectivity in the presence of both APOBEC3G and Vif.** VSV-G pseudo-typed luciferase-reporter virus was produced in the presence or absence of co-transfection of APOBEC3G expression vector. Before harvesting virus-containing supernatant, 10 μM MM-1 or MM-2 were added to culture medium. Harvested supernatant was challenged to fresh 293 T cells, and luciferase activity of lysates was measured after two days. Samples of virus and producer cells were also analyzed by immunoblotting. APOBEC3G levels in the virus particles were recovered by treatment of the compounds.

We next asked whether the small molecules we found actually impair viral infectivity by using luciferase-reporter virus. 293 T cells were transfected with NL4-3 ΔEnv-Luc with co-transfection of a VSV-G expression vector, in the co-transfection of an APOBEC3G expression vector, and treated with MM-1 or MM-2, or DMSO as control. NL4-3 ΔEnv ΔVif-Luc was also used for comparison. Supernatants were harvested and challenged to fresh 293 T cells. The infectivity of *vif*-deficient virus was deeply impaired by co-transfection of APOBEC3G, and the infectivities of *vif*-proficient virus in the presence or absence of APOBEC3G were comparable as expected (Figure 3, histogram). Treatment with MM-1 or MM-2 recovered sensitivity of the virus to APOBEC3G (Figure 3, histogram). Immunoblotting analyses showed recovery of APOBEC3G levels in both producer cells and in virus particles by treatment of MM-1 or MM-2 (Figure 3, panels).

### The small molecules did not inhibit ubiquitination of APOBEC3G by HIV-1 Vif

**Figure 4:**
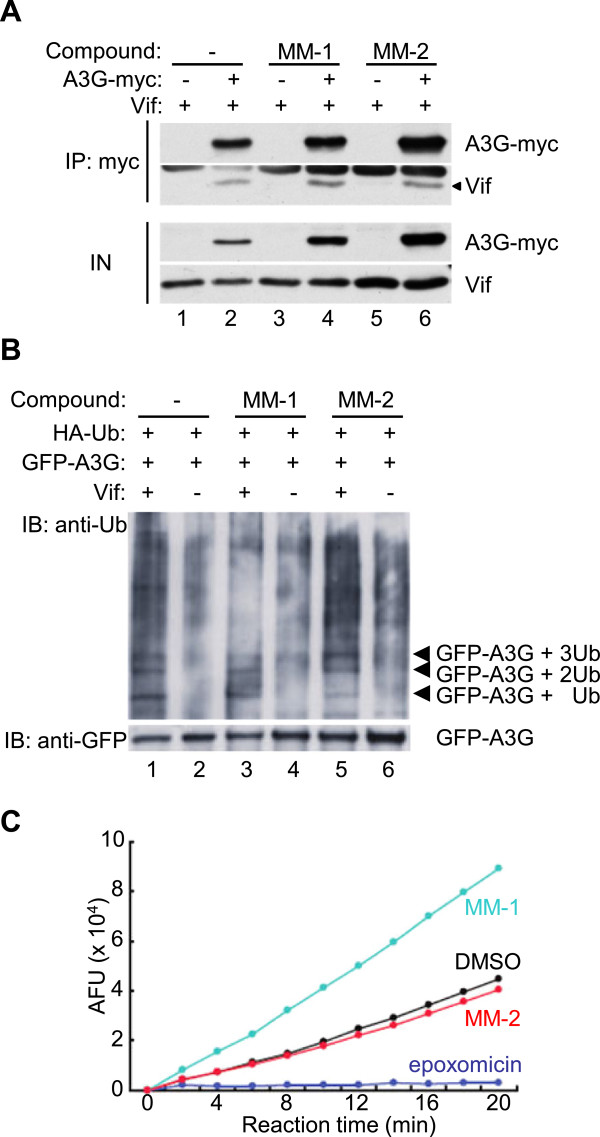
**The small molecules did not inhibit ubiquitination of APOBEC3G or general proteasomal activity. A.** 293 T cells were transfected with the Vif expression vector, co-transfected with APOBEC3G-myc expression vector or empty vector. 24 hours before harvesting, MM-1, MM-2 or DMSO was added in culture media as indicated. Cells were lysed and immunoprecipitated by anti-myc antibody in the presence of the compound as indicated, and bound protein was analyzed by immunoblotting with anti-Vif and anti-myc sera. **B.** 293 T cells were transfected with expression vectors for EGFP-fused APOBEC3G and HA-tagged ubiquitin, in the absence or presence of co-transfection of the Vif expression vector, and treated with MM-1, MM-2 or DMSO for 24 hours. Cells were then lysed, and immunoprecipitated with anti-GFP rabbit serum. Bound protein was analyzed by immunoblotting with anti-ubiquitin and anti-GFP antibodies. **C.** An *in vitro* proteasome 20S assay kit was used for testing inhibitory potential of the compounds to proteasomal activity. DMSO was used as control for the compounds and epoxomicin was used as control for proteasome inhibitor.

To determine what step of the process the candidate small molecules interfere, we first examined binding between APOBEC3G and Vif in the presence or absence of the small molecules. 293 T cells were co-transfected with expression vectors for Vif and carboxyl-terminally myc-tagged APOBEC3G or empty vector, lysed with 0.1% triton X-100 containing buffer, and immunoprecipitated with a monoclonal anti-myc antibody, with or without addition of MM-1 or MM-2 in both cell culture and the lysis buffer. MM-1 or MM-2 did not appear to inhibit co-precipitation of Vif protein (Figure 4A), suggesting that neither molecule inhibits binding between APOBEC3G and Vif. We next examined ubiquitination of APOBEC3G by Vif in the presence or absence of the small molecules. 293 T cells were transfected with the expression vector for EGFP-APOBEC3G, with or without co-transfection of the Vif expression vector, treated with MM-1 or MM-2, or DMSO as control, immunoprecipitated with anti-GFP antibody, then analyzed for ubiquitination of APOBEC3G by immunoblotting with anti-ubiquitin antibody. Co-transfection of Vif resulted in several ubiquitinated APOBEC3G with higher molecular weights, and treatment with MM-1 or MM-2 appeared to make no difference in these bands (Figure 4B). These results suggest that MM-1 or MM-2 does not inhibit Vif-mediated ubiquitination of APOBEC3G, and are consistent with the results of co-immunoprecipitation experiments. We next examined whether the candidate small molecules inhibit proteasomal activity by using an *in vitro* 20S proteasome assay kit, which can measure chymotrypsin-like peptidase activity of purified human erythrocyte 20S proteasome. MM-1 or MM-2 was added in the reaction to test inhibitory effects, DMSO was used as control for the small molecules, and epoxomicin was used as a proteasome inhibitor. Addition of MM-1 or MM-2 did not impair 20S proteasome activities (Figure 4C), suggesting that recovery of APOBEC3G by these compounds are not simply caused by inhibiting the general proteasome activities.

### Cytotoxicity studies of the compounds

**Figure 5:**
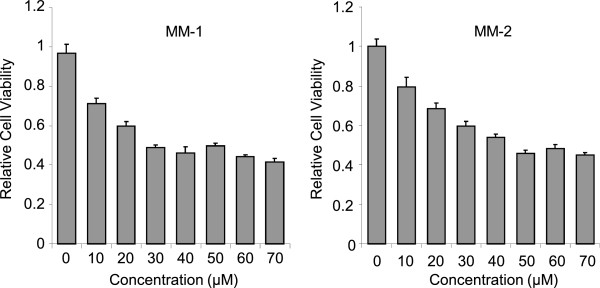
**Cytotoxicity of the small compounds.** 293 T cells were treated with MM-1 or MM2 at indicated concentrations for 48 hours. Cell viability was measured by MTS assay and normalized to that of DMSO-treated cells. Average and standard error of six independent experiments are shown.

We next examined cytotoxic effects of the candidate small molecules. 293 T cells were incubated with various concentrations of MM-1, MM-2 or DMSO for 48 hours, and cell viability was then measured by MTS assays. We observed significant cytotoxicity of both molecules at 10 μM or higher concentrations, and IC_50_ of MM-1 was about 30 μM and that of MM-2 was about 50 μM (Figure 5).

## Discussion

We have used multi-round of screening based on APOBEC3G reporter systems and identified two candidate small molecules for inhibiting Vif-mediated degradation of APOBEC3G. We confirmed that the small molecules recover APOBEC3G levels in producer cells by immunoblotting analyses as well as fluorescence measurement, and also confirmed that these molecules recover incorporation of APOBEC3G into *vif*-proficient viruses. Our data support the idea that inhibition of Vif-mediated degradation of APOBEC3G by small molecules can suppress HIV-1 replication.

Nathans *et al.* reported 25 candidate small molecules which recover APOBEC3G expression levels in the presence of Vif by using YFP- and RFP-fused APOBEC3G protein [20]. However, none of these molecules have structural similarity to the molecules we found, likely because different libraries and/or different screening methods were used. Our study therefore provides another class of candidate Vif inhibitor compounds for further development.

In the immunoprecipitation analyses, we did not observe any change in ubiquitination of APOBEC3G by treatment of MM-1 or MM-2, nor did we observe any changes in co-immunoprecipitation experiments. These results suggest that the small molecules do not interfere with the Vif-APOBEC3G interaction. Moreover, the compounds did not inhibit general proteasomal activity. Therefore, the small molecules we identified might work in the step between ubiquitination of APOBEC3G by Vif and proteasomal degradation of ubiquitinated APOBEC3G. Considering that the molecules we identified appeared to up-regulate APOBEC3G levels in cells even in the absence of Vif protein, the molecules might bind to APOBEC3G and make it more stable even after ubiquitination. Because we used a cell-based screen that relies upon fluorescence of EGFP-fused APOBEC3G, candidate molecules may inhibit any step of the entire process by which APOBEC3G is degraded. Additional screens will be necessary to identify small molecules that directly block the Vif-APOBEC3G interaction.

Of the two molecules we identified, MM-2 was more effective for causing a recovery in APOBEC3G levels as well as the restriction of HIV-1, and it appeared to be less toxic to 293 T cells. However, because cytotoxicity of the small molecules we identified is observed at concentrations very close to the concentration which these molecules are effective, the molecules are not likely to become drugs for patients with HIV-1 infection. However, considering the fact that these two small molecules share a similar chemical backbone, derivatives with similar core structures might become candidates for further development.

## Conclusions

We have validated the concept that inhibiting Vif-mediated degradation of APOBEC3G can result in restricted HIV-1 replication. In addition, we have added another structural class to the growing library of candidate Vif-inhibiting small molecules. Derivatives of the small molecules we identified might become candidates for further development.

## Methods

### Plasmids

The expression vectors for EGFP-fused APOBEC3G and Vif were previously described [21,22]. The expression vector for HA-tagged APOBEC3G (pcDNA3/HA-A3G) was previously described [23]. The expression vector for luciferase-fused APOBEC3G was generated by inserting coding sequence of luciferase gene amplified with primers NNN NGC TAG CGC CAC CAT GGA AGA CGC CAA AAA CAT and NNN NCT CGA GCA CGG CGA TCT TTC CGC CCT at Nhe I and Xho I sites of pcDNA3/HA-A3G. pNL4-3/ΔEnv-Luc and pNL4-3/ΔenvΔVif-Luc vectors were previously described [21].

### Cell culture and transfection

293 T cells were maintained in DMEM (Nacalai) containing 10% FBS and 1% penicillin–streptomycin and glutamine (PSG, Invitrogen). CEM and CEM-SS cells were maintained in RPMI1640 containing 10% FBS and 1% PSG. 293 T cells on 6-well plates were transfected with about 1 μg of plasmid DNA in total using X-tremegene HP DNA transfection reagent (Roche) according to manufacturer’s instruction.

### Screening for small molecules that inhibit Vif-mediated degradation of APOBEC3G

A library of small compounds was purchased from Enamine. For the primary screening, 293 T cells on 24-well plates were transfected with expression vectors for EGFP-APOBEC3G and Vif and treated with individual molecules from a library of small compounds for 24 hours, then cells were lysed with M-PER (Pierce), and fluorescence intensity was measured by plate reader (2030 Arvo X, Perkin Elmer). DMSO was used for control for the compounds and cells transfected with only EGFP-APOBEC3G were used for positive control for fluorescence intensity. Experiments were performed in duplicate and repeated once more, and the compounds repeatedly recovered fluorescence intensity to more than 50% were selected to secondary screening. For the secondary screening, 293 T cells on 24-well plates were transfected with expression vectors for Luc-APOBEC3G and Vif and treated with compounds for 24 hours, Passive lysis buffer (Promega) and luciferase activity was determined by luminometer (2030 Arvo X, Perkin Elmer) using Luciferase Assay System (Promega). DMSO was used for control for the compounds and cells transfected with only Luc-APOBEC3G were used for positive control. Experiments were performed in duplicate and the compounds which recovered chemiluminescence intensity to more than 50% were selected for tertiary screening. For the tertiary screening, CEM and CEM-SS cells were challenged with NL4-3 at 0.01 MOI and treated with the compounds for 10 days. The compounds which rescued virus-inducing cell death of CEM cells, but not that of CEM-SS cells were selected.

### Immunoblotting

Cells were lysed with triton-based buffer (20 mM HEPES-HCl pH7.4, 150 mM NaCl, 1 mM EDTA, 0.1% triton X-100) supplemented with protease inhibitor cocktail (Nacalai). After centrifugation at 20, 000 × g for 10 minutes, supernatant was mixed with sample buffer (Biorad), boiled for 5 minutes, resolved on 10% (w/v) polyacrylamide gel and transferred to PVDF membrane (Immobilon, Millipore). The membrane was blocked for non-specific binding of antibodies with 5% BSA containing buffer for 1 hour and analyzed by standard immunoblotting procedure. Primary antibodies for immunoblotting against Vif, A3G and p24^Gag^ were obtained from the NIH AIDS Research and Reference Reagent Program. Mouse anti-tubulin antibody was purchased from Covance. Rabbit anti-GFP serum was purchased from Invitrogen. Mouse anti-ubiquitin monoclonal antibody was purchased form Santa Cruz. HRP-conjugated secondary antibodies against mouse and rabbit were purchased from GE Healthcare.

### Infectivity studies

Luciferase encoding HIV-1 particles were produced by transiently transfecting 293 T cells at 50% confluency using 0.6 μg pNL43/ΔEnv-Luc or pNL43/ΔEnvΔVif-Luc, 0.15 μg pVSV-G and 0.25 μg pcDNA3/HA-A3G, or an empty vector. After 48 hours, virus-containing supernatants were harvested through PVDF filter with 0.45 μm pores (Millipore), and challenged to fresh 293 T cells. Medium were changed after 12 hours, cells were incubated for additional 36 hours, lysed with Passive lysis buffer (Promega) and luciferase activity was determined by luminometer (2030 Arvo X, Perkin Elmer) using Luciferase Assay System (Promega). Sample preparation of producer cells and virus for immunoblotting was performed as described [24].

### Immunoprecipitation

For co-immunoprecipitation, 293 T cells were transfected with the expression vectors for Vif, with co-transfection of the expression vector for APOBEC3G-myc or empty vector. Cells were treated with 10 μM of MM-1, MM-2 or DMSO only for 24 hours and 2.5 μM MG132 for 16 hours, then washed with PBS, lysed with co-IP buffer (25 mM HEPES, pH 7.4, 150 mM NaCl, 0.1% triton X-100, 1 mM EDTA, 1 mM MgCl_2_, and 10% glycerol) supplemented with protease inhibitor cocktail and 10 μM MG132, centrifuged for 10 minutes at 20,000 × g. Supernatant was incubated with anti-myc mouse monoclonal antibody (clone 9E11) for 1 hour, and then mixed with 20 μl protein A sepharose (Pharmacia) for 1 hour. Beads were washed with co-IP buffer three times, and bound protein was eluted with 1 × SDS sample buffer.

For testing ubiquitination of APOBEC3G, 293 T cells were transfected with expression vectors for HA-tagged ubiquitin and EGFP-fused APOBEC3G, with or without co-transfection of the expression vector for Vif. Cells were treated with 2.5 μM MG132 for 16 hours before harvest, then washed with PBS, lysed with RIPA buffer (25 mM HEPES, pH 7.4, 150 mM NaCl, 0.1% SDS, 0.1% sodium deoxycholate, 1% triton X-100, 1 mM EDTA) supplemented with protease inhibitor cocktail and 10 μM MG132, centrifuged for 10 minutes at 20,000 × g. Supernatant was incubated with anti-GFP rabbit serum (Invitrogen) for 1 hour, and then mixed with 20 μl protein A sepharose (Pharmacia) for 1 hour. Beads were washed with RIPA buffer three times, and bound protein was eluted with 1 × SDS sample buffer. Samples were analyzed by immunoblotting as described above.

### Proteasomal activity studies

Proteasome 20S assay kit (Enzo) was used according to manufacturer’s instruction. 10 μM of MM-1 or MM-2 was added to the reaction, and DMSO and epoxomicin are used as controls.

### Cytotoxicity studies

293 T cells were incubated with various concentration of MM-1 or MM-2 for 48 hours, and cell viability was measured by MTS assays using a kit (Promega). Cell viability was normalized to that of cells incubated with the same concentration of DMSO.

## Competing interests

The authors declare that they have no competing interests.

## Authors’ contributions

MM performed most of the experiments and wrote the manuscript. KS participated in virus incorporation and cytotoxicity studies, and wrote the manuscript. TI, KI and MS participated in the screening. JK designed the experiments and participated in the screening. MK designed experiments and wrote the manuscript. NK wrote the manuscript. RSH wrote the manuscript. ATK designed the experiments and wrote the manuscript. All authors read and approved the final manuscript.
